# ‘You’re on the waiting list’: An interpretive phenomenological analysis of young adults’ experiences of waiting lists within mental health services in the UK

**DOI:** 10.1371/journal.pone.0265542

**Published:** 2022-03-18

**Authors:** Georgia Punton, Alyson L. Dodd, Andrew McNeill

**Affiliations:** Department of Psychology, University of Northumbria at Newcastle, Newcastle-Upon-Tyne, United Kingdom; Newcastle University, UNITED KINGDOM

## Abstract

Waiting lists in mental health services are currently considered too long. Lengthy waits of up to 18 weeks are commonly reported in the UK. Waiting lists have long been associated with a variety of negative psychological responses, however there is little understanding behind young adults’ personal experiences of such delays within mental health services. The current study aimed to explore young adults’ experiences of waiting lists in mental healthcare in the UK. Seven young adults were interviewed in the current study (aged 19–22). Interpretive phenomenological analysis was utilised to explore participant accounts. Three super-ordinate themes were generated: Reliance on Alternative Methods of Support; Inability to Function Sufficiently; and Emergence of Negative Beliefs, Emotions and Thoughts. Participants primarily reported a variety of negative psychological and behavioural consequences associated with waiting lists in mental health services, as well as exacerbated existing physical and psychological health issues. In accordance with the limited number of previous studies, waiting lists are considered by patients to be barriers to mental health support and intervention. Future direction is advised.

## Introduction

Waiting lists organise and co-ordinate numbers of people requiring a service that is in high demand. Due to a lack of funding and resources, demand for the UK’s National Health Service (NHS) is growing, which has led to longer waiting lists [1, 2]. Concerns have emerged regarding how this will impact the physical and psychological wellbeing of patients, especially in mental health services where waiting lists remain consistently long [3].

Within mental healthcare systems, long waiting lists have been observed for accessing psychological therapies, such as counselling or cognitive behavioural therapy (CBT) [4]. To address these concerns, NHS England implemented waiting list standards in 2016 as part of the Improving Access to Psychological Therapies (IAPT) programme, with the target of 75% of referrals for psychological interventions to be assessed and treated within six weeks, and 95% within 18 weeks [5, 6]. Despite a recent IAPT report suggesting that these standards are being met [7], ‘hidden’ delays for treatment sessions still remain extensive, with 50% of overall referrals in certain areas of the UK waiting for up to 162 days (23 weeks) to receive both an initial consultation and a secondary follow-up session [4]. Furthermore, over 100,000 patients who require treatment withdraw from this programme before completion each year, and such attrition within mental health referrals has been considered to be linked to these longer waits for treatment and between sessions [3]. This is consistent throughout the UK, with more than a third of Scottish child and adolescent mental health service (CAMHS) patients and a fifth of adult mental health service (AMHS) patients waiting longer than the 18-week target [8, 9]. Similar unmet target times have been observed in Northern Ireland and Wales [10].

Regardless of new guidelines, waiting lists in mental health services continue to be described as ‘too long’ for those with mental health issues due to their vulnerability and need for immediate care [10, 11]. These long waits are often reported by patients directly, who state that waiting lists act as a barrier to help-seeking across both CAMHS and AMHS [12, 13]. Long waiting lists are also faced by those transitioning from CAMHS to AMHS [14]. Individuals within this transition period face hardship at this pivotal stage, including feelings of abandonment, struggling to cope without continued care, and ‘not feeling ready’ to move onto AMHS [15], which may be exacerbated by these long waits.

Student populations seeking support from university mental health services also report long waiting lists [16]. Students are often signposted to these services prior to, or during NHS referral, and may assume they will be seen sooner than other services. However, many university services have poor staffing ratios and lack sufficient funding to meet demand [17].

Waiting lists have been associated with negative psychological and physiological responses such as anxiety and stress; more uncertain, unexpected and longer waits lead to further aggravation of these symptoms [18, 19]. Such effects are exacerbated among those awaiting health-related news or treatment due to the potentially severe implications of delays [20]. Within general healthcare, patients waiting for diagnosis, surgery or treatment experience increases in negative affect, including anger, frustration, fear, stress, anxiety and depression, as well as reduced self-esteem, which worsened the longer they waited [20, 21].

These effects are mirrored within patients awaiting mental healthcare, where reports state that perceived uncertainty and lack of support due to longer waits led to reduced functioning, prolonged emotional distress, exacerbation of existing symptoms [22] and poorer outcomes across a range of domains [23]. Some findings suggest higher rates of suicide or suicide-related hospitalisations [24], emphasising the serious implications long waiting lists could have upon those with mental health issues.

Additionally, longer waits have also been associated with greater drop-out and disengagement once intervention begins [25], possibly reflecting a lack of trust in the service and reduced motivation to participate [26]. In turn, poor therapeutic engagement is associated with mental health deterioration and a greater chance of future hospital admission [27]. Excessive waits in public services also lead to greater demand for private intervention for access to support sooner [13, 28, 29].

While there is some evidence to support the barriers to mental healthcare and the implications of not receiving said care [30] there is little empirical research which specifically investigates the consequences of waiting lists for these services in the UK from the perspective of those facing them. Young adults aged 18–25, who are seeking mental health support from AMHS or IAPT, may be particularly affected by lengthy waiting times. Young adults face new responsibilities and experiences as independence and a sense of self is established. Such transitions in life can be difficult, and have been linked to decline in mental health and wellbeing [31]. Considering that the age of first onset for many mental health disorders is within this young adult demographic [32], it is important to understand how waiting lists may influence help-seeking and engagement with UK services.

Therefore, the current study aimed to explore the experience of facing waiting lists for mental health services by those who have recently sought psychological intervention in the UK. By employing a qualitative design, the objectives of this research are to understand the consequences of these delays by investigating individualistic experiences using Smith’s [33] interpretive phenomenological analysis (IPA). The research questions were:

1. How do young adults seeking psychological interventions make sense of their experiences of waiting lists in mental health services?
2. How do young adults cope with being on a waiting list for mental health services?

## Methods

### Research design

IPA was applied for a rigorous, double-hermeneutic exploration of participant’s understanding of their own experiences [34] as informed by Heidegger’s phenomenology [35]. This method is widely used in health psychology research due to its idiographic emphasis of participant’s phenomenological experience, as well as the acknowledgement of the importance of the researcher’s reflexive interpretation [34]. To balance these requirements while exploring this lived experience, the researcher approached IPA from a contextualist position as characterised by critical realism.

### Participant sample and recruitment

Purposeful sampling was employed to ensure adherence to the inclusion criteria, which were: (1) to have experienced a waiting list when seeking psychological mental health support lasting at least six weeks, aligning with IAPT’s guidelines of waiting list standards [5]; (2) experienced this wait within the last three years; (3) be aged between 18 and 25. Recruitment was conducted using posters and leaflets placed around a university campus and through social media posts. Interested individuals were sent an online screening questionnaire to ensure demographic information and eligibility (e.g., ‘how long were your waiting for mental health treatment from time of referral?’, ‘how long ago were you referred?’).

A final screened sample of seven participants was obtained. Smaller, more concise samples are common in IPA studies to allow for more in-depth interpretation, rather than superficial understanding which may accompany a more diverse sample [33]. This sample consisted of five women and two men. They were all aged between 19 and 21 during their wait (*mean age* = 20), and between 19 and 22 at the time of interview (*mean age* = 20.5) and all waiting lists lasted between 6 and 47 weeks (*mean length* = 16 weeks). All participants were students at university and were located in the North East of England, West Midlands or Scotland during their wait. Varying mental health concerns were represented, including anxiety, depression, bipolar disorder, and OCD. Across the sample, participants were waiting for a range of interventions, including counselling, CBT and psychiatric review.

Peer-interviewing was employed, as the primary researcher (GP) was of a similar age and a student at a UK university with training in conducting semi-structured interviews.

### Data collection procedure

Ethical approval was obtained from the Northumbria University Undergraduate Psychology Ethics Committee (ref: 12288). An initial pilot interview was conducted to evaluate the initial interview protocol, observe the depth of the discussion, and identify any redundant questions or changes required. This interview was not included in the final data.

Potential participants were asked to provide informed consent and then complete an online survey asking for demographic information (e.g., age, sex and gender identity) and to screen for their eligibility. Individual, in-person interviews were then arranged and conducted in a private room at a university. Consenting participants were asked a total of seven open-ended questions (i.e., ‘Why did you initially seek professional mental health support?’; ‘Can you tell me about the delays or waits that you faced?’; ‘How did this delay of support impact your life?’; ‘Would you say this delay in support had any kind of positive impact?’; ‘How did you end up coping during this time?’; ‘If you were to experience another waiting list for mental healthcare, how might you approach it?’; ‘What do you think could be done to assist with the growing issue of long mental health waiting lists?’). The structure of the interview was flexible, however, numerous prompts and probes were implemented within each interview to assist with the collection of detailed data. Participants were debriefed and provided information for both university and UK mental health services in case of lingering distress (e.g., University Nightline Services, Samaritans, Mind). Interviews lasted between 32 and 50 minutes (*mean length* = 37 minutes).

Audio recordings of the interviews were transcribed verbatim by the researcher to allow for the maintenance of context and meaning of the participant’s experience [34]. Names or identifying features were redacted for anonymity. Recordings were revisited to correct any initial transcription errors.

### Data analysis procedure

Data were analysed using a flexible application of IPA [36]. The aim for the analysis was to generate, categorise and organise any potential themes as observed within the data while carefully considering the unique experiences of each participant. Firstly, transcriptions were read while simultaneously listening to the original audio recording to allow for complete immersion in the participant’s narrative. Individual transcripts were analysed by annotating and commenting on the dialogue and its semantic and latent content. Comments and annotations were then analysed to identify potential codes, emergent themes, and connections for each individual transcript, before ordering these themes into preliminary associated clusters charted based on their prominence. Initial themes were then further organised into succinct secondary emergent themes, and finally, three higher-order super-ordinate themes. To ensure rigour and respondent validation, all participants were contacted for a member check to evaluate how accurately they felt the final themes portrayed their experiences. Responses from all seven participants led to minor adjustments and a final classification of three super-ordinate and five sub-ordinate themes, and these were verified once again by participants to accurately reflect the overall experiences. Therefore, the final themes were not included based solely on prevalence within the data and attempted to encompass the rich, diverse and complex experiences from the perspective of the participants [36].

### Role of the researcher

At all stages, the researcher (GP) aimed to engage in reflexive practice and implemented strategies to ensure quality and trustworthiness throughout data collection, analytical interpretation and within the general methodology. Firstly, the interview questions were continuously scrutinised and peer-reviewed by a senior researcher (AD). Additionally, constructive feedback and direction on the analysis procedure was provided by a senior researcher (AM), and final themes were also peer-reviewed and challenged (AD). Additional commentary was sought from participants via member checking as to validate findings among the sample themselves [37, 38]. Such feedback allowed for credibility and rigour to be ascertained across researchers [39], while also prioritising the voices of the participants. Primarily, it was important to certify that the analytic process did not remove the initial context derived from lived experience from the final themes. A common phenomenological practice to mitigate this is bracketing, where a researcher separates from their own preconceptions and understanding of a phenomena before engaging with participant accounts [40]. However, being interpretive in nature, IPA does not require, nor assume fully reductive focus, and therefore, the researchers attempted to balance this technique with personal interpretation through the use of empathetic and open reflection upon preconceptions and biases, without bracketing them entirely [35, 41]. Finally, as a peer, the primary researcher had insight to understand the life-circumstances of participants and how mental health interacted with their lives.

## Findings

Three super-ordinate themes were formed during the analysis, which all participants expressed in some form: Reliance on Alternative Methods of Support (T1); Inability to Function Sufficiently (T2); and Emergence of Negative Beliefs, Emotions and Thoughts (T3). Two of these super-ordinate themes overarched five overall sub-ordinate-themes (Table 1).

**Table 1:** Super-ordinate and sub-ordinate themes generated using IPA.

Themes and Codes			
Thematic Level	Theme One	Theme Two	Theme Three
**Super-Ordinate Themes**	T1. Reliance on Alternative Methods of Support	T2. Inability to Function Sufficiently	T3. Emergence of Negative Beliefs, Emotions and Thoughts
**Sub-Ordinate Themes**	Seeking Alternative Intervention Development of Coping Mechanisms Reliance on Social Support	Decline in Mental State and Existing Symptoms Impact Upon Lifestyle & Physical Ability	

### Reliance on alternative methods of support (T1)

Reliance on alternative methods of support (T1) was formed as a super-ordinate theme in response to the lack of support available while on a waiting list. All participants expressed a need for various forms of additional help while waiting.

#### Seeking alternative intervention (T1.a)

Nearly all participants sought alternative intervention (T1.a) while waiting for intervention. This mainly consisted of additional psychological treatment or medication.

*‘I was able to come to terms a lot more with a lot of the stressful things that I wouldn’t have been able to do without the medication*, *but I still think therapy would have been more beneficial to me’*

–Beth (woman, 21).

*‘I initially started on anti-anxiety medication*, *which overall did have a positive effect*, *but I was very reluctant to at the time… the sense of not having help*, *especially due to not being able to afford it*, *not being able to access the free stuff makes you feel lost*, *or that you don’t have any help available’*

–Luke (man, 20).

While participants disclosed that medication was effective in reducing their symptoms, some shared that this was often different to the psychological intervention they felt was right for them initially. This coincides with previous findings, which have found that some people might take medication or another form of treatment because their preferred form of intervention is not available [29, 42]. Luke states that he felt lost with no help available, which could explain why he ended up opting for anti-anxiety medication, despite initial reluctance; his need for intervention surpassed the desire to avoid pharmaceuticals, and conveyed desperation for improvement in his mental health regardless of intervention. Furthermore, Luke’s reference to feeling lost given his inability to access the ‘free stuff’ highlights the constrains of his economic situation and how this impacts his perception of managing his mental health. He sees ‘the free stuff’ as the only option and the only alternative is to ‘feel lost’, or to take any available alternative. The growing body of help-seeking literature states that many with mental health concerns avoid interventions until their situation becomes desperate and they are no longer able to manage it themselves [43, 44] and given the current strain on mental health services, medication might be the only immediate viable option. This might also influence participants’ decision to try other routes.

*‘they said to me at the doctors the waiting list is so long it’s probably best to start with your uni and see your uni wellbeing team… the thing is you can get like six [sessions] per semester*. *It’s not enough… sometimes you’re waiting quite a few weeks*, *whereas sometimes they’ll be like oh we’re free tomorrow’*

–Hannah (woman, 22).

*‘I decided to go private due to worsening symptoms*, *in part due to the waiting list [12–18 weeks]*, *in part due to just mental health situation… I enjoyed it but it quickly became too expensive for me to sustain*.’

–Luke.

Hannah recalls the doctors advising her to seek help from external sources. Hearing this from official, trusted sources of support conveys helplessness and uncertainty from percieved support providers over the duration of the waiting lists, and prompted her to seek alternative support. Free alternative services, such as university mental health services, also tend to be limited in session availability and continue to have issues with inconsistent long waits due to high student demand [16]. Additionally, longer waiting lists in public healthcare are associated with greater purchases of private therapies and insurance [28, 45]. However, private therapies are costly, especially for young adults, who might not have a permanent career or who are students in higher education.

While there are alternative interventions for young adults, these are more percieved as temporary solutions with quicker access to sustain individuals before they are granted psychological support.

#### Development of coping mechanisms (T1.b)

Some participants also developed coping mechanisms (T1.b) while placed on waiting lists. Both adaptive and maladaptive behaviours were disclosed.

*‘I took up meditation as well… that definitely helped me relax and kind of take a step back and chill out’*–

Matt (man, 20).

*‘ I really want to get into yoga because I know that will help*, *I know that’s really good for your mind and I really want to get into mindfulness and meditation*. *I used to do that for chronic back pain and it did help’*

–Hannah.

Some participants relied on coping mechanisms such as mindfulness and meditation while waiting for further intervention; Hannah showed an awareness that engaging in these practices would be beneficial, and related this to previously coping with physical health concerns, although was yet to implement them. Such techniques are standard and effective when managing stress and anxiety independently [46], and have also allowed individuals in adverse situations to cope more adaptively and constructively, boasting long-lasting benefits in a variety of healthcare settings [47]. It is not surprising that individuals would adopt such techniques to assist during the wait for mental healthcare, however, depending on the mental health concern, some people might find mentally engaging in adaptive practices difficult, despite knowing their potential benefit [48].

An additional finding was expressed by participants who held the perception that their background knowledge of their circumstances allowed them to understand and deal with the corresponding symptoms.

*‘as a psychology student*, *you understand that it’s just an irrational thought’*

–Lucy (woman, 21).

*‘being like a psychology student*, *it’s so easy to be like*, *this is what I need*, *but I wanted them to tell me what I need rather than to tell myself’*

–Hannah.

Lucy and Hannah’s reflection on their experience supports past literature, which has found that psychology students with personal experience of mental health issues have high mental health literacy [49]. Mental health literacy, has been seen to improve help-seeking and promote the development of adaptive coping mechanisms [50, 51]. Therefore, perhaps those who have a deeper understanding of their issues as attained through their academic or personal life can manage such concerns more appropriately. Despite this insight, Hannah still expressed a need for validation of her experiences from an authoritative figure, suggesting that the acquisition of knowledge alone is likely inadequate. To futher this point, Lucy did manage to attain some reassurance and clarification from a professional in the form of a diagnosis.

*‘someone said*, *I think you have like generalised anxiety; I was like right I’m not going mad… the wait did me some good but maybe like I say*, *not that long of a wait*.*’*

–Lucy.

She found her diagnosis helped her to rationalise that she was not ‘going mad’. However, before participating in CBT, Lucy stated that she needed time to process her recent diagnosis of general anxiety disorder. Diagnostic labelling can lead to feelings of relief and elation for some, as those seeking support receive professional acknowledgement and justification for their disorder [52]. The diagnosis itself helps patients to cope and further allows for a brief period of self-reliance before further intervention is required. Yet, it is important to note that some might perceive a diagnosis as unhelpful, given that negative stereotypes might lead to self-stigmatisation and lowered self-esteem, which can facilitate self-fulfilling effects in the form of worsening symptoms [53]. Additionally, the waiting list was still too long for Lucy, suggesting that prompt treatment should still be prioritised.

Not all coping mechanisms were constructive, with many participants taking on more destructive behaviours.

*‘I was so unwell in my head*, *that it became oh*, *well I need to become bad so that they believe me*, *they need to see how bad it is*. *So*, *there was a lot that pushed me to self-harm during that time… it definitely added to the destructive nature of my [bipolar] disorder because there was so much frustration and you either take it out on other people or on yourself*, *and I took it out on myself*.*’*

–Amy (woman, 20).

Self-harm is consistently linked to other mental health issues such as depression, bipolar disorder and schizophrenia [54], however, the delay that Amy was facing further exacerbated the destructive tendencies of her disorder. From her perspective, this was for both the purpose of releasing frustration, but to also express the critical nature of her situation to those who could grant her help (see T3.a). Those with mental health disorders are at much higher risk of self-harming as a coping mechanism [54], therefore participants’ frustration combined with delayed intervention seemed to exacerbate self-harm risk and lead to physical and mental detriment. Similar to Hannah, Amy also sought professional validation and reassurance, but further stresses this ‘need to be believed’. Given her desperation for support and her distress at the time, she percieved self-harm as a viable way to be attain help in a maladaptive form of help-seeking, or as a ‘cry for help’ [55]. Self-harm is also reported as a way for individuals to maintain control over a situation [56], and because waiting (particularly for health-related support) is considered an uncontrollable circumstance [20, 21], it could be interpreted that Amy’s use of self-harm was to regain a sense of agency in an increasingly uncertain and distressing situation.

*‘I turned to smoking marijuana and drinking alcohol quite frequently… that was the worst point of my mental health decline because I didn’t have any help*.*’*

–Luke.

For Luke, maladaptive coping also manifested in the form of substance use. These methods were potentially approached as short-term solutions, especially as substance use has been considered a likely coping mechanism throughout mental health literature [57]. Qualitative research has found that perceived barriers to support, such as access to services, might encourage substance use as a substitute for professional intervention in students [58]. As those with mental health disorders display a pre-existing vulnerability to maladaptive strategies [59], this demonstrates the urgency for those with psychiatric disorders to receive mental healthcare, lest they reinforce destructive methods of coping, especially in such high-risk cases as bipolar disorder.

#### Reliance on social support (T1.c)

All seven participants expressed their reliance on social support during the delay (T1.c) by depending on family, friends and others to provide psychological support.

*‘I needed the support sooner rather than later*, *and I’m lucky that I had friends and family and stuff there for me’*

–Beth.

*‘My friends especially helped in ways that I wasn’t expecting them too*. *I knew my girlfriend very well and that she would be supportive*, *but I didn’t realise there was a big support structure in my friends*. *Once that was established it was a lot easier to deal with and I ended up*, *despite being on the waiting list*, *not needing intervention*.*’*

–Luke.

Social support has been consistently reported as a means for people to manage their symptoms and mental health issues [60]. Literature continuously finds that those with better social support systems, or who receive greater attentiveness from close relations suffer from less mental distress [61]; social support commonly acts as a buffer which can moderate the development of both mental and physical disorders [62]. Interestingly, while Beth was aware of the support she had available from her friends and family, Luke was unaware of his support structure in the beginning. Research has found that while women are relatively open with their mental health needs among their peers, men report having difficulties in reaching out for social support [63], particularly among other male peers, and thus, might only disclose their emotional needs during times of distress [64]. Yet, once established, Luke found that this reliance on his friends seemed to contribute towards his eventual withdrawal from further treatment from public services, supporting the need for fostering positive support systems to help mitigate the impact of long waits. Particularly, Lucy looked to her close relations to reframe her thinking.

*‘My Dad’ll go more of the logical way around it*, *whereas I’m really emotional-based*, *so I’ve gone with*, *no think about how maybe my dad would say it and how he’d see it*.*’*

–Lucy.

By observing her father’s processing style, she was able to comprehend her anxieties and rationalise them before they escalated. Social observation is considered a way for those with mental health issues to understand their own experiences, through modelling and imitating the behaviours of others and adapting them as their own [65]. Therefore, Lucy was able to conduct this social learning mechanism based on her father’s behaviour to help her manage her irrational thoughts while she was waiting for psychotherapy. This could suggest that close relations are relied on for more than just support during delays, but to also help amend negative thinking patterns or aberrant behaviours.

Finally, an important observation was the repercussions that the waiting list had upon the family and friends of those seeking help.

*‘While I was on the waiting list I wasn’t in anybody’s care… it became the responsibility of the people around me… the people who put me on the waiting list didn’t say to my family*, *oh*, *okay so this is what you need to do in the meantime*, *they were just totally left*.*’*

–Amy.

Amy describes how the delay negatively affected her family, as they were required to care for her due to the lack of intervention from services. The families of those with mental health issues commonly report that they receive little support from mental health services and are usually left to deal with the situation without guidance [66], suggesting that the consequences of waiting lists extend beyond the individual seeking the help, to also affecting the wellbeing of close relations. This shifting of responsibility from service to family in these times of uncertainty might also facilitate further problems for the individual, as young people especially seem to see themselves as a burden to those close to them [67]. By stating ‘the people who put me on the waiting list’, Amy percieves that those who referred her have capacity to do something and that capacity should be employed in equipping others, even if they themselves are unable to help.

### Inability to function sufficiently (T2)

The second super-ordinate theme presented an inability to function sufficiently (T2). This illustrates the concern that delays in mental health support interfered with day-to-day life, as well as physical and mental functioning.

#### Decline in mental state and existing symptoms (T2.a)

It was reported that delays in mental health treatment seemed to exacerbate existing symptoms of participant’s mental health issues (T2.a). This was expressed by all participants, with varying degrees of severity. One of the more common experiences was heightened anxiety and stress levels due to the uncertainty surrounding the delay and its length.

*‘It certainly worsened the anxiety for me personally because I felt more alone and more vulnerable to my own mind… it’s not knowing that there’s any definitive end to it*.*’*

–Luke.

Previous research suggests that uncertainty is commonly associated with stress, anxiety and nervousness especially when applied to healthcare scenarios [21]; therefore, it is understandable that participants might experience a decline in mental health, or at least a shift from acute to chronic symptoms over time. All participants expressed their struggle with anxiety or anxiety-related symptoms while waiting, suggesting that this uncertainty might exacerbate existing mental health concerns.

*’They’ve sort of come back with the response of like*, *you’re not bad enough*, *and I think that’s like*, *so dangerous because it pushes people to think*, *like*, *they’re not taking me seriously*.*’*

–Hannah.

*‘I was told ‘you’re not severe enough’… hearing you’re not bad enough when you’re at your absolute lowest it really just messes with your mind and you think okay*, *I’m not bad enough*, *time to get bad enough*, *so that’s why I would self-harm and that’s where the suicidal thoughts come in and like… just anything to be able to say at my next appointment*: *I did this*, *am I bad enough yet*? *I wanted to present from the outside how I felt on the inside*. *So*, *I stopped looking after myself physically*.*’*

–Amy.

Additionally, participants stated that their worsening symptoms were due to perceptions of the services not believing their issues were severe enough to be seen sooner. Hannah and Amy again illustrate the impact of an authoritative voice, however, for Amy in particular being told that she were not severe enough for immediate treatment facilitated destructive behaviours with the intent of being seen quicker. For those suffering from mental health problems, this damaging experience accompanied by being delayed from mental health intervention is likely to bring about self-fulfilling behaviours intended to express the severity of mental state and bring about further decline to be taken seriously and receive treatment, either by adopting maladaptive coping mechanisms (T1.b) or by externalising how they feel mentally.

### Impact upon lifestyle and physical ability (T2.b)

Almost all participants expressed that the delays they faced had a substantial impact upon their everyday activities, lifestyle and obligations (T2.b). This was mainly conveyed by their difficulty studying, attending lectures and committing to academic requirements.

*‘Because it took so long to get on the waiting list I then had to move back home… I was no longer safe to be left by myself and then I had to defer uni because the care took so long to get into place… my GP didn’t always have appointments and when they did*, *I was just told*, *you’re on the waiting list there’s nothing more we can do*.*’*

–Amy.

Amy made it clear she left university due to the delay of psychological support, as she was no longer safe to be left on her own without observation. While past research has found that students with mental health issues are more likely to drop out of higher education [68], what ultimately influenced her decision to leave university and move back home was her inability to single-handedly manage on the waiting list, despite continuously attempting to receive help. This left her vulnerable and needing to seek help from her family (T1.c), and highlights the disadvantage experienced by those with mental health issues.

*‘the reason I wanted the help is because it’s my final year of uni… I want to aim as high as I can but I feel like it’s holding me back because I’m not getting help*.*’*

–Hannah.

Participants regularly stated that their mental health issues caused them to feel more unmotivated, stressed and disconnected in terms of their university experience, and further indicated that the delays they were facing exacerbated these issues and caused greater complications with academic success. Previous research has suggested that mental health issues and associated stressors are associated with poorer educational enjoyment, productivity and academic performance [68]. Therefore, further stress caused by these waiting lists for psychological intervention could be presumed as yet another obstacle impeding academic success, progress and fulfilment.

In addition to university, many participants expressed issues with completing everyday tasks and basic functioning.

*‘I had to call in sick for work sometimes*. *I couldn’t eat*, *I couldn’t shower*, *I just couldn’t physically get out of bed which just stopped me doing everything’*

–Luke.

*‘I didn’t shower*, *I didn’t get out of my bed because I thought in my head I thought maybe if I stay in bed long enough someone will come and section me and then I’ll get help’*

–Amy.

Luke expressed helplessness as he was unable to physically undergo any of his normal everyday activities, as is diagnostically common in those with mental health disorders. He found that these issues grew more prominent due to the waiting list he was placed on, again suggesting that delays in mental healthcare might not directly cause these negative outcomes but exacerbate them. This was also presented by Amy, however, her inability to function seems to be driven by the desire to be provided with treatment as soon as possible. As mental health services tend to prioritise more serious cases [69], by neglecting to physically care for herself, Amy was aiming at being seen as a priority. She further explained the desperation behind her actions and expressed that her irrationality was triggered by the waiting list. And so, uncertain delays can influence irrational help-seeking behaviour which is detrimental to an individual’s physical and mental functioning.

Additionally, some participants also expressed the impact the delays had upon their pre-existing physical conditions as well as their mental health.

*‘I’ve got stress incontinence and arthritis and they’re always worse when I’m stressed… my arthritis flared up*, *so I was struggling*.*’*

–Beth.

*‘I’m quite anaemic*, *my iron is really depleted at the minute because I’ve not been eating as well*, *so that makes me more tired and less motivated than I was*, *so I think it’s like a chain reaction’*

–Hannah.

Stress brought on by the delay in treatment caused both Beth and Hannah’s health issues to deteriorate to the point where their everyday function was impaired. Hannah likens this relationship to a ‘chain reaction’, which perhaps suggests that the delay in treatment triggers corresponding negative effects upon both her mental and physical health. The contents of these extracts are supported by the mass of literature signifying the strong association between stress, health problems and physical decline, with long-term stressors being associated with a variety of physical issues [70]. Therefore, being exposed to the long-term stress of waiting lists may cause further decline in pre-existing physical health conditions.

### Emergence of negative beliefs, emotions and thoughts (T3)

The final super-ordinate theme formed was the emergence of negative beliefs, emotions and thoughts (T3) participants experienced while waiting for treatment. Participants commonly developed negative beliefs and readily described the strong emotions that they felt as a result of the delays.

*‘I feel more anger towards it than anything else*. *Some people will be like at long last*, *but for me it’s like*, *it’s about bloody time*.*’*

–Alex.

*‘It does put a strain on a lot of things to know that some medical conditions can be seen so quickly*, *whereas it almost seems as though mental health isn’t classed as the same sort of severity as physical*.*’*

–Hannah.

Alex assessed their own anger as stronger than that of hypothetical others in the same situation. Comparison is a common way for those experiencing hardships to express the extent of their negative experiences [71] and is likely a way to convey desperation for the anticipated psychological intervention. As Alex also held the expectation that the waiting time was to be shorter, this could also have exacerbated their frustration. These expectations also seem to play a role in the emotional response of Hannah; previous experiences of timely care for physical health issues meant that she had similarly high expectations when seeking mental health support. This resulted in disappointment, and inevitably frustration, when this expectation was not met. This is described well by Jeronimus and Laceulle [72], who state that frustration “is elicited when a goal-pursuit is not fulfilled at the expected time in the behavioural sequence (an unexpected non-reward)” (p. 01). It is only understandable that those seeking mental health treatment are left frustrated when they initially assume prompt support. When these expectations are not met, it may also influence feelings or states of despair, or hopelessness.

*‘On the actual day of my suicide attempt it was like*, *I’m never going to get help*, *it’s going to be like this forever*.*’*

–Amy.

Amy’s suicide attempt was seemingly associated with the belief that there was no end to the constant delays, and she would never receive intervention. Intrusive feelings of hopelessness are thought to be a risk factor for self-injury and suicidal behaviour, especially when paired with irrational beliefs [73]. Therefore, the negative emotions and beliefs which manifest due to these delays could be reinforcing harmful behaviours in those requiring mental health support. Feelings of hopelessness and corresponding irrational beliefs were reported consistently by all participants.

*‘I didn’t deserve the help when I knew that I needed it*.*’*

–Beth.

*‘I’m never going to get help so why help myself*?*’*

–Alex.

This interaction between situation, belief and emotion can be explained by the ABC model of irrational beliefs [74], where an *activating event*, (i.e., being placed on a waiting list), could lead to a corresponding *belief* (i.e., ‘I’m not bad enough’), and then to an emotional *consequence* (i.e., hopelessness or frustration). However, it has been suggested that irrational beliefs are external representations of biased schemas formed by individuals based on their subjective experiences [75]. These schemas can become increasingly prominent if a particular event or situation is considered detrimental, stressful, or if it occurs repeatedly; they can also influence negative affective appraisals, causing emotional responses to be somewhat contradictory given the reality of the situation [76]. Therefore, individuals experiencing a long waiting list for mental health treatment might develop or reinforce existing schemas, form irrational beliefs regarding their worth or their situation and appraise their emotions more negatively. Such irrational beliefs and emotions have been suggested to influence behavioural responses which may contribute to suicidal tendencies, the notion of ‘getting worse to get help’ or the development of maladaptive coping mechanisms [77].

### Methodological considerations and limitations

The findings of the current study should be approached within the context of its methodological limitations. Despite not being an inclusion criterion, all participants recruited were students during their wait. Students make up a large proportion of the UK population between the ages of 18–25 [78], and have been found to be at a higher risk of developing mental health disorders than the general population [79]. Given that students are a highly specific demographic, it is important that this sample is broadened in future research to allow for the understanding of the consequences of these waiting lists among other groups. For example, it would be of value to explore the experiences of young adults outside of higher education. Unlike students, this group does not have access to additional institutional mental health support services which may act as a temporary buffer while waiting for intervention, and they also display different social support structures [80]. Considering these disparities in service access and existing support, it will be important to investigate this population to establish a more comprehensive understanding of young adult’s experiences.

This study also implemented peer-interviewing, where the researcher was of similar age and circumstance to the participants (an undergraduate student). While the researcher consciously approached each interview with impartiality, it is important to acknowledge the potential problems that may have influenced participant responses, including bias, over disclosure by participants, or conversely, concern over judgements [81]. However, considering the sensitive content of this research, having existing rapport with participants likely fostered empathy and encouraged a more open and honest discussion [82]. Additionally, the primary researcher underwent interview training and continuously engaged in reflective exercises to help mitigate these issues and improve the quality of the data attained [81].

### Implications and future direction

The current study has provided a preliminary understanding into the consequences of mental healthcare delays as experienced on an individual level. These findings currently sit among the foundations of this body of literature, and so, further research should continue with both qualitative and quantitative enquiry of the impacts of waiting lists within mental healthcare. One potential avenue could investigate the potential implications of individual differences within those on waiting lists. The present study indicated that waiting lists in mental healthcare are associated with a variety of negative consequences, including the development of maladaptive coping mechanisms and the emergence of negative or irrational beliefs. However, some participants expressed greater issue with the waiting lists than others, which may be due to individual differences, such as mental resilience, social support and lifestyle. There is clearly a need for more quantitative enquiry to explore potential factors that might influence individual’s attitudes and help-seeking behaviours when long waits are present in mental healthcare.

Finally, the implications of these findings, although preliminary, align with the current body of literature which points towards a need for systemic development in the accessability and delivery of mental health services [29, 83, 84], particularly concerning further support provisions for those experiencing such delays.

## Conclusion

The current study identified several detrimental consequences for young adults awaiting support for their mental health. While some participants attempted to reflect upon their experiences in a constructive manner, this was not without criticism. Furthermore, delays in treatment were found to exacerbate existing mental and physical health symptoms, and attempts to cope with these long waits ranged from adaptive to maladaptive strategies. Seeking out alternative forms of intervention was also reported, and young adults might opt for medication, private or university services to get support quicker and help sustain them while waiting for psychological therapies. Young adults also placed importance on seeking professional opinion and validation from authority figures in relation to their issues, even if they were aware and knowledgable of their symptoms. As increasing demand of mental health services leads to longer waiting lists, the current findings illustrate the need for systemic change, particularly concerning further support provisions for those experiencing delays for mental healthcare. Future research is also needed to understand how waiting lists might impact different populations.

## Supporting information

S1 File Supplementary materials, including interview schedules, full analytic procedure, theme generation and a copy of the consolidated criteria for reporting qualitative research (COREQ) can be found at: https://osf.io/cafhd/. (DOC)
